# Adiposity Is Associated with Gender-Specific Reductions in Left Ventricular Myocardial Perfusion during Dobutamine Stress

**DOI:** 10.1371/journal.pone.0146519

**Published:** 2016-01-11

**Authors:** Michael E. Hall, Tina E. Brinkley, Haroon Chughtai, Timothy M. Morgan, Craig A. Hamilton, Jennifer H. Jordan, R. Brandon Stacey, Sandra Soots, W. Gregory Hundley

**Affiliations:** 1 Department of Medicine, Division of Cardiology, University of Mississippi Medical Center, Jackson, Mississippi, United States of America; 2 Department of Medicine, Section on Gerontology and Geriatric Medicine, Wake Forest School of Medicine, Winston-Salem, North Carolina, United States of America; 3 Division of Cardiology, Beloit Memorial Hospital, Beloit, Wisconsin, United States of America; 4 Department of Biostatistical Sciences, Wake Forest School of Medicine, Winston-Salem, North Carolina, United States of America; 5 Department of Biomedical Engineering, Wake Forest School of Medicine, Winston-Salem, North Carolina, United States of America; 6 Department of Medicine, Section on Cardiovascular Medicine, Wake Forest School of Medicine, Winston-Salem, North Carolina, United States of America; Virginia Commonwealth University Medical center, UNITED STATES

## Abstract

**Background:**

Obesity and visceral adiposity are increasingly recognized risk factors for cardiovascular disease. Visceral fat may reduce myocardial perfusion by impairing vascular endothelial function. Women experience more anginal symptoms compared to men despite less severe coronary artery stenosis, as assessed by angiography. Women and men have different fat storage patterns which may account for the observed differences in cardiovascular disease. Therefore, our objective was to evaluate the relationship between visceral adipose tissue distributions and myocardial perfusion in men and women.

**Methods:**

Visceral and subcutaneous fat distributions and myocardial perfusion were measured in 69 men and women without coronary artery disease using magnetic resonance imaging techniques. Myocardial perfusion index was quantified after first-pass perfusion with gadolinium contrast at peak dose dobutamine stress.

**Results:**

We observed inverse relationships between female gender (r = -0.35, p = 0.003), pericardial fat (r = -0.36, p = 0.03), intraperitoneal fat (r = -0.37, p = 0.001), and retroperitoneal fat (r = -0.36, p = 0.002) and myocardial perfusion index. Visceral fat depots were not associated with reduced myocardial perfusion at peak dose dobutamine in men. However, in women, BMI (r = -0.33, p = 0.04), pericardial fat (r = -0.53, p = 0.02), subcutaneous fat (r = -0.39, p = 0.01) and intraperitoneal fat (r = -0.30, p = 0.05) were associated with reduced myocardial perfusion during dobutamine stress.

**Conclusions:**

Higher visceral fat volumes are associated with reduced left ventricular myocardial perfusion at peak dose dobutamine stress in women but not in men. These findings suggest that visceral fat may contribute to abnormal microcirculatory coronary artery perfusion syndromes, explaining why some women exhibit more anginal symptoms despite typically lower grade epicardial coronary artery stenoses than men.

## Introduction

Obesity is associated with multiple cardiovascular and metabolic derangements including hypertension, diabetes, dyslipidemia, endothelial dysfunction, and inflammation that have been linked to the development of coronary artery disease and heart failure. Visceral adiposity is recognized to play an important role in the development of many of the metabolic disorders which increase the risk for developing coronary artery disease (CAD).

Pericardial fat is associated with severity of CAD [1, 2]. It has been hypothesized that fat around the coronary arteries may trigger an inflammatory state resulting in endothelial dysfunction [3]. Visceral abdominal adiposity is associated with markers of peripheral vascular endothelial dysfunction whereas subcutaneous fat is not [4]. From these studies it appears that accumulation of fat in compartments other than subcutaneous tissues has adverse effects on the vasculature although the effects on coronary artery blood flow and left ventricular myocardial perfusion have not been extensively studied.

Women are more likely than men to experience angina despite typically having less obstructive coronary artery disease on angiography [5]. It has been hypothesized that this finding is related to microvascular disease [6] and endothelial dysfunction [7, 8]. Differences in fat distributions between men and women may account for some of the clinical differences observed in manifestations of cardiovascular disease.

The objective of this study was to evaluate the associations between visceral adiposity and myocardial perfusion during inotropic myocardial stress with dobutamine in men and women. To accomplish these goals, cardiac magnetic resonance (CMR) evaluation of myocardial perfusion during dobutamine infusion was quantitated and compared with MR measures of pericardial, abdominal subcutaneous, intraperitoneal, and retroperitoneal fat volumes.

## Materials and Methods

### Study population

This study was approved by the Institutional Review Board (IRB) at Wake Forest School of Medicine and all study participants provided written, witnessed informed consent. Four hundred and forty-one consecutive participants from the National Institutes of Health funded cohort study, “Pulmonary Edema and Stiffness of the Vascular System” (PREDICT), were enrolled to evaluate vascular system abnormalities that predict future cardiac events. Middle aged and elderly individuals (aged 55–85 years) with risk factors for an incident cardiovascular event were recruited to receive a CMR exam followed by semiannual surveillance for cardiovascular events. Participants were ineligible for enrollment if they had a contraindication for CMR (implanted metal, pacemakers, defibrillators, other electronic devices or claustrophobia), active acute coronary, cerebral, or peripheral vascular symptoms, severe aortic stenosis or other significant valvular disease, uncontrolled tachyarrhythmias, or a contraindication to the receipt of gadolinium contrast (estimated glomerular filtration rate <45 mL/min/1.73m^2^). For the present analysis, participants were also excluded if they had evidence of myocardial infarction based on self-reported history, positive findings on late gadolinium enhancement images or if there were obvious areas of inducible ischemia that were visible on stress perfusion images. For this substudy analysis, 69 participants were included.

### Study design

Each participant underwent a history, physical exam, laboratory evaluation and a comprehensive CMR imaging exam upon enrollment. Body mass index (BMI) was calculated from measured height and weight. Afterward, participants underwent a CMR exam at a field strength of 1.5T (Siemens Avanto scanner, Erlangen, Germany). During the CMR exam, images for the purpose of determining abdominal fat were acquired according to previously published techniques [9–11]. Dobutamine stress CMR was also performed on each participant. Dobutamine was infused incrementally from low dose (7 to 10 microgram/kg/min, i.v.) to high dose (20 to 40 micrograms/kg/min, i.v.), and atropine was administered to achieve 80% of the maximum predicted heart rate response for age, the heart rate response associated with a maximal test [12, 13]. Perfusion images were acquired at rest and high dose dobutamine infusion. Late gadolinium enhancement images were obtained 10 minutes after the second bolus of gadolinium contrast.

### Image Analysis

**Fig 1:**
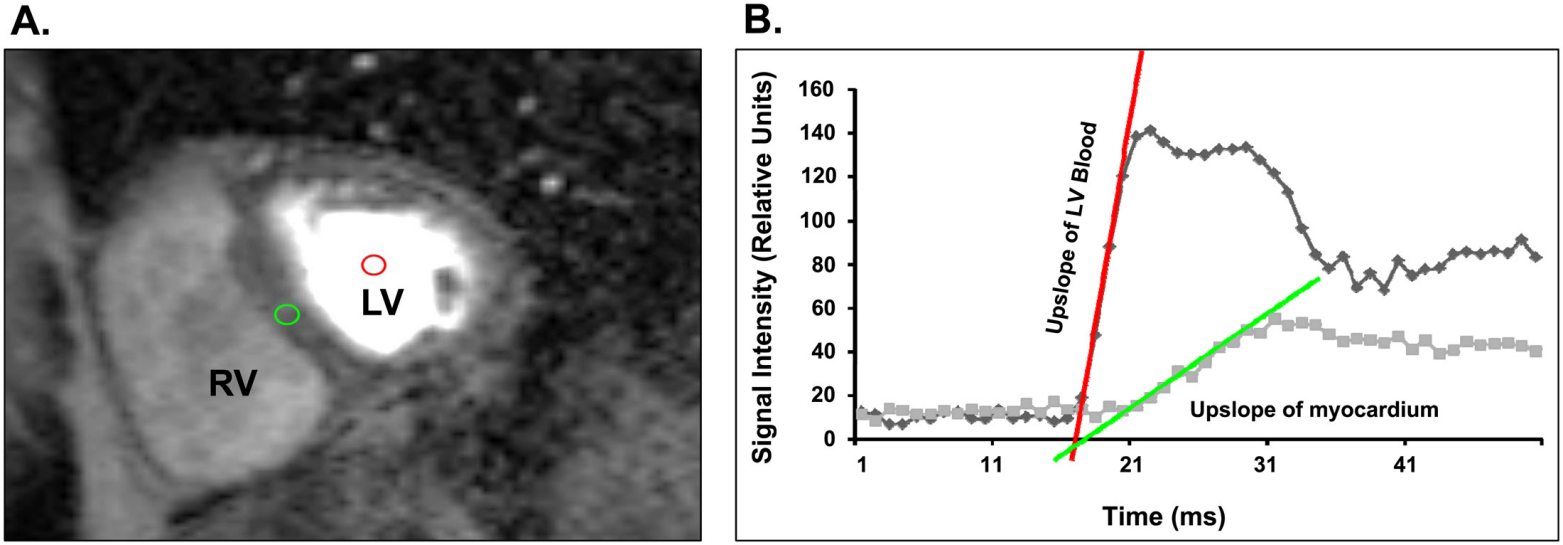
Measurement of myocardial perfusion index. (A) Segment from representative mid-ventricular slice during first-pass perfusion imaging during peak dose dobutamine stress. Red region of interest defines the middle of the left ventricle (LV) for measuring the slope of blood, and the green circle defines the middle interventricular septal myocardium which was used to measure the slope of myocardial perfusion. (B) The signal intensities (Y-axis) over time (X-axis) were plotted for blood (red) and LV myocardium (green). The upslope of LV blood and upslope of LV myocardium were plotted and used for determination of myocardial perfusion index (MPI).

Abdominal and pericardial fat volumes were determined according to previously published techniques [9–11]. Also according to previously published techniques [14], myocardial perfusion index (MPI) was calculated after first-pass perfusion with gadolinium contrast at peak dobutamine stress. Regions of interest (ROIs) were drawn on at least 50 phases of the perfusion images by a single board certified cardiologist with expertise in CMR. A ROI was drawn in the center of the left ventricular cavity at a middle ventricular short-axis slice to quantitate left ventricular arterial input. A second ROI was drawn on the same middle ventricular slice except it was placed in the middle interventricular septum as this segment is less susceptible to cardiac motion (Fig 1A). Both ROIs were then propagated and adjusted manually as necessary through all segments to ensure similar positioning. MPI was calculated as the upslope of signal intensity over time plot for left ventricular myocardium divided by the upslope of signal intensity over time plot for the left ventricular blood (Fig 1B) [14]

### Statistical analysis

All data are presented as mean ± standard error. Two-tailed Students t-tests were performed to evaluate the differences in men and women. Pearson’s correlation coefficients were determined to evaluate the associations of age, gender, race, diagnosis of hypertension or diabetes, segmental fat depots, and BMI with MPI. For all tests, a p-value <0.05 was considered significant.

## Results

### Participant characteristics

Clinical characteristics of the study participants according to gender are shown in Table 1. The mean age of the 69 participants evaluated was 65±0.8 years. Seventy percent of the participants were white and 61% were women. The mean BMI of the participants was 30.8±0.8 kg/m^2^. Thirty-nine percent were diabetic and 93% were hypertensive.

**Table 1:** Comparison of characteristics between women and men.

	Women (n = 42)	Men (n = 27)	p-value
Age (mean, years)	65.6 ± 6.9	65.1± 7.2	0.68
Race (% White)	71	67	0.77
BMI (kg/m^2^)	30.6 ± 6.8	31.1 ± 4.5	0.74
Diabetes (%)	29	56	0.03*
Hypertension (%)	95	89	0.33
Ejection Fraction (%)	59.5 ± 3.3	58.2 ± 3.4	0.09

BMI = Body mass index,

* denotes statistical significance

There were no significant differences in age or race between men and women (p = 0.77 and p = 0.68 respectively). BMI was similar in men and women (p = 0.74) and there was no significant difference in diagnosis of hypertension between men and women (p = 0.33). However, diabetes was more prevalent in men compared to women (p = 0.03).

### Myocardial perfusion and left ventricular parameters in men versus women

MPI at the peak dose of dobutamine was higher in women compared to men (0.20 ± 0.01 vs 0.15 ± 0.01, p = 0.002). There were no significant differences in LV ejection fraction in women compared to men (59.5 ± 0.5% vs 58.2 ± 0.7%, p = 0.10).

### Fat volumes

Despite similar BMI measures between women and men, men generally had more visceral fat compared to women (Table 2). Women tended to have more subcutaneous fat than men, although the differences were not statistically significant (p = 0.18).

**Table 2:** Fat depot volumes in men and women.

Fat depot	Women	Men	p-value
Subcutaneous	246.1 ± 124.2	206.6 ± 111.9	0.18
Intraperitoneal	134.5 ± 66.3	234.9 ± 89.9	0.33
Retroperitoneal	35.0 ± 17.9	69.0 ± 24.3	<0.001*
Pericardial	70.2 ± 36.8	103.6 ± 54.6	0.04*

Fat volumes were quantitated as cm^3^,

* denotes statistical significance

### Myocardial perfusion and fat relationships

Associations of MPI and covariates age, race, sex, diagnosis of diabetes or hypertension, BMI, and fat volumes (pericardial, subcutaneous, intraperitoneal and retroperitoneal fat) were assessed using Pearson’s correlation coefficients. Female sex (p = 0.003), pericardial fat (p = 0.02), intraperitoneal fat (p = 0.002) and retroperitoneal fat (p = 0.003) were inversely associated with MPI (Table 3).

**Table 3:** Correlations between covariates and myocardial perfusion index at peak dose dobutamine in both men and women.

	Pearson correlation coefficient (r)	p-value
Age	0.21	0.08
Race (white)	0.04	0.72
Sex (female)	-0.36	0.002*
Diabetes	-0.11	0.36
Hypertension	-0.04	0.72
BMI (kg/m^2^)	-0.25	0.06
Pericardial fat	-0.39	0.02*
Subcutaneous fat	-0.18	0.12
Intraperitoneal	-0.38	0.002*
Retroperitoneal fat	-0.36	0.003*

* denotes statistical significance

Further sex-specific relationships were evaluated. In men, no visceral fat depots were associated with reduced MPI at peak dose dobutamine stress (Table 4). However, in women, BMI (p = 0.04), pericardial fat (p = 0.02), subcutaneous fat (p = 0.01), and intraperitoneal fat (p = 0.05) were associated with reduced myocardial perfusion at the peak dose of dobutamine (Table 4).

**Table 4:** Sex-specific correlations between covariates and myocardial perfusion index at peak dose dobutamine.

p-value	Women		Men	
	Pearson correlation coefficient (r)	p-value	Pearson correlation coefficient (r)	
Age	0.24	0.12	0.17	0.40
Race (white)	-0.04	0.82	0.39	0.04*
Diabetes	0.03	0.83	-0.15	0.46
Hypertension	-0.18	0.27	0.03	0.88
BMI (kg/m^2^)	-0.33	0.04*	0.14	0.51
Pericardial fat	-0.53	0.02*	-0.13	0.61
Subcutaneous fat	-0.39	0.01*	0.10	0.61
Intraperitoneal	-0.30	0.05	-0.12	0.56
Retroperitoneal fat	-0.18	0.25	-0.20	0.31

* denotes statistical significance

## Discussion

We investigated the relationships between visceral and subcutaneous fat volumes and impaired LV myocardial perfusion at peak dose dobutamine stress in asymptomatic adults with no known history of CAD or previous myocardial infarction. We observed a negative correlation between visceral adipose tissue volumes and peak dose dobutamine myocardial perfusion. Although women had higher MPI compared to men overall, higher fat volumes were associated with impaired LV stress myocardial perfusion in women but not in men. While men had higher visceral fat volumes compared to women, our findings suggest that women may be more susceptible to the detrimental effects of visceral adiposity on stress-induced measures of LV myocardial perfusion. Several previous studies have documented impaired myocardial blood flow in response to vasodilator stimuli such as adenosine suggestive of coronary microvascular dysfunction. To our knowledge, this is the first study demonstrating an association between impaired myocardial perfusion at with inotropic stress and visceral adipose tissue in women.

Our findings are consistent with other published studies showing associations with visceral adiposity and impaired myocardial perfusion [15]. However, most of these studies were performed using adenosine or regadenoson stress while the current study was performed with dobutamine stress. Dobutamine causes inotropic stimulation of the heart through β-adrenergic activation to increase the workload of the heart whereas adenosine and regadenoson cause coronary vasodilation and myocardial hyperemia. Normally, the heart is adapted to meet its energetic requirements at moderate levels of physiologic stress [16]. It has been demonstrated that catecholamine stress further exacerbates the energetic deficit in obesity measured by phosphocreatinine to adenosine triphosphate levels leading to impaired myocardial relaxation in obese individuals compared to normal-weight subjects [16]. The authors postulated the mechanisms of this metabolic deficit were reduced mitochondrial oxidative phosphorylation or inadequate blood supply to the heart during stress related to microvascular dysfunction. A limitation of this study was the lack of myocardial perfusion imaging. Our findings of reduced myocardial perfusion during dobutamine stress support microvascular dysfunction as a potential cause of energetics and functional derangements during stress in obesity.

Our results may help to explain why women have more angina symptoms compared with men despite typically less severe epicardial coronary vessel stenosis [6]. In a study of type 2 diabetic patients with CAD amenable to revascularization (BARI 2D study), women were more likely than men to have angina despite less severe coronary artery disease as assessed by angiography [6]. In the BARI 2D study, men and women received similar medication regimens and had similar rates of death, myocardial infarction, and cerebrovascular accidents. The authors postulated that the relative increase in angina symptoms in women compared with men with more epicardial CAD burden may be attributed to endothelial dysfunction. Furthermore, impaired coronary vascular reactivity has been associated with adverse outcomes in women [17].

Both obesity and female gender are independent predictors of higher myocardial blood flow and oxygen consumption [18]. Despite greater cardiac work, myocardial efficiency is less in women than in men and this may be related to impaired glucose utilization and insulin resistance. This may explain, at least in part, why myocardial perfusion is negatively associated with visceral fat in women but not in men.

Associations between visceral obesity and endothelial dysfunction in both the coronary and peripheral arterial circulations have been described [19, 20]. This relationship may be related to the adverse effects of obesity on insulin resistance, dyslipidemia, hypertension, and vascular inflammation [21]. Modest weight gain (average of 4.1kg) resulted in impaired endothelial function even in the absence of changes in blood pressure. Endothelial dysfunction was predicted by visceral fat, not subcutaneous fat, and recovered after weight loss [22]. However, in our study, both visceral fat (pericardial and intraperitoneal) and subcutaneous fat were associated with reduced MPI. Further studies evaluating the mechanisms by which specific fat depots may mediate myocardial perfusion should be performed. In women the strongest relationship was between pericardial fat and impaired myocardial perfusion. The relationship between pericardial fat and impaired cardiac perfusion has been described and may be related to local inflammatory cytokine production [15]. Although fat volume, in general, may contribute to systemic inflammation, our findings suggest that fat in closest proximity to the myocardium and coronary arteries (ie pericardial fat) may have the most detrimental effect on myocardial perfusion. We did not observe a significant relationship between pericardial fat and CRP levels (data not shown), however it is possible that local paracrine effects of fat may lead to local coronary artery inflammation instead of systemic inflammation which we measured with CRP. Further studies will need to be performed to determine if greater pericardial fat in one coronary artery territory negatively impacts myocardial perfusion in the same territory. Our findings are consistent with other published data. In a study of 68 women with angina and no obstructive CAD, epicardial fat was an independent predictor of reduced coronary flow reserve, a marker of coronary microvascular dysfunction [23].

Our participant population was relatively aged (mean 65 years) and women were likely post-menopausal. Endothelial dysfunction appears to develop during the early stages of menopause and progressively worsens with prolonged estrogen deficiency [24, 25]. Visceral adiposity increases and serum estradiol levels tend to decrease in women after menopause [26]. The cardiovascular effects of reduced estrogen levels in postmenopausal women are not completely understood. Estrogen receptors are present on vascular endothelium and estrogen stimulates the release of nitric oxide and causes vascular smooth muscle relaxation [25, 26]. Conversely, decreased levels of estrogen may increase the activity of endothelin-1 and norepinephrine which could contribute to changes in myocardial perfusion during stress.

The relationship between visceral adipose tissue and estrogen is not well understood, but the enzyme 17β- hydroxysteroid dehydrogenase type 1, which catalyzes the conversion of cortisone to cortisol, is highly expressed in the visceral fat of postmenopausal women and is associated with reduced estradiol levels [27]. In obese men, sex steroid levels (estradiol and testosterone) do not depend on visceral fat volumes. However, in obese women visceral fat loss results in a reduction in androgenicity.

Results of recent studies suggest that visceral adiposity may play a key role in the development of ischemic heart disease and congestive heart failure. Much of this risk may be attributed to the concomitant known risk factors for heart disease such as hypertension, diabetes and dyslipidemia, or concomitant comorbidities including inflammation and endothelial dysfunction. The effect of visceral adiposity in relation to gender is less well established but it seems that visceral adipose tissue may be particularly harmful in aged women. This is important because obesity is more prevalent in women compared with men, and after menopause there is often increasing visceral adiposity [28]. Metabolic syndrome is associated with a higher risk of cardiovascular death in women than in men and the metabolic syndrome is more prevalent in women after menopause [29, 30].

Our study has some limitations. This is a cross-sectional study so a direct cause-and-effect relationship between visceral adiposity and impaired myocardial perfusion cannot be determined. However, our findings are consistent with the hypothesis that visceral fat adversely affects the heart. Although we hypothesize that visceral fat may impair coronary blood flow due to endothelial dysfunction, we did not measure endothelial function in this study. Other studies have linked visceral obesity with impaired vascular reactivity, however, data evaluating the effects of sex and hormonal differences in this setting are understudied. The participants evaluated in this study presumably were free of significant, flow-limiting epicardial coronary stenosis but they did not undergo coronary angiography for verification. Overall, the number of study participants (particularly males) was relatively low and this may impact the power of some of our findings. Also, the majority of our participants were white so this may affect generalizability.

We excluded all participants with visually apparent perfusion defects which would suggest significant coronary arterial stenoses and participants with delayed enhancement suggestive of previous myocardial infarction. While our findings demonstrate a significant relationship between impaired myocardial perfusion and visceral adiposity in women only, this does not suggest that visceral adiposity in men is benign. In fact, visceral adiposity and derangements that accompany it such as hypertension, diabetes and dyslipidemia, are well established risk factors for epicardial coronary disease in both men and women. Our findings shed light on a potential risk factor for small vessel coronary disease which has been attributed to chest pain syndromes in women with normal epicardial vessels. Epicardial coronary vasculature in men may be more sensitive to the adverse effects of visceral fat and concomitant metabolic factors.

## Conclusions

Visceral fat is associated with impaired myocardial perfusion at peak dose dobutamine. There is a sex-specific effect suggesting impaired endothelial function in women with increased adiposity. The exact mechanisms by which fat contributes to reduced myocardial perfusion have not been completely elucidated and need to be studied in future experiments.
